# Targeted delivery of MerTK protein via cell membrane engineered nanoparticle enhances efferocytosis and attenuates atherosclerosis in diabetic ApoE^−/−^ Mice

**DOI:** 10.1186/s12951-024-02463-y

**Published:** 2024-04-13

**Authors:** Shuo Qiu, Jiahan Liu, Jianmei Chen, Yangni Li, Te Bu, Zhelong Li, Liang Zhang, Wenqi Sun, Tian Zhou, Wei Hu, Guodong Yang, Lijun Yuan, Yunyou Duan, Changyang Xing

**Affiliations:** 1 Department of Ultrasound Medicine, Tangdu Hospital, Air Force Medical University, No.569, Xinsi Road, Xi’an, 710038 China; 2 Department of Health Medicine, The Fourth Medical Center of Chinese PLA General Hospital, Beijing, China; 3 The State Key Laboratory of Cancer Biology, Department of Biochemistry and Molecular Biology, Air Force Medical University, Xi’an, China

**Keywords:** Hybrid membrane, Efferocytosis, Atherosclerosis, Diabetes, MerTK

## Abstract

**Background:**

Clearance of apoptotic cells by efferocytosis is crucial for prevention of atherosclerosis progress, and impaired efferocytosis contributes to the aggravated atherosclerosis.

**Results:**

In this study, we found that diabetic ApoE^–/–^ mice showed aggravated atherosclerosis as hyperglycemia damaged the efferocytosis capacity at least partially due to decreased expression of Mer tyrosine kinase (MerTK) on macrophages. To locally restore MerTK in the macrophages in the plaque, hybrid membrane nanovesicles (HMNVs) were thus developed. Briefly, cell membrane from MerTK overexpressing RAW264.7 cell and transferrin receptor (TfR) overexpressing HEK293T cell were mixed with DOPE polymers to produce nanovesicles designated as HMNVs. HMNVs could fuse with the recipient cell membrane and thus increased MerTK in diabetic macrophages, which in turn restored the efferocytosis capacity. Upon intravenous administration into diabetic ApoE^–/–^ mice, superparamagnetic iron oxide nanoparticles (SMN) decorated HMNVs accumulated at the aorta site significantly under magnetic navigation, where the recipient macrophages cleared the apoptotic cells efficiently and thus decreased the inflammation.

**Conclusions:**

Our study indicates that MerTK decrease in macrophages contributes to the aggravated atherosclerosis in diabetic ApoE^–/–^ mice and regional restoration of MerTK in macrophages of the plaque via HMNVs could be a promising therapeutic approach.

**Graphic Abstract:**

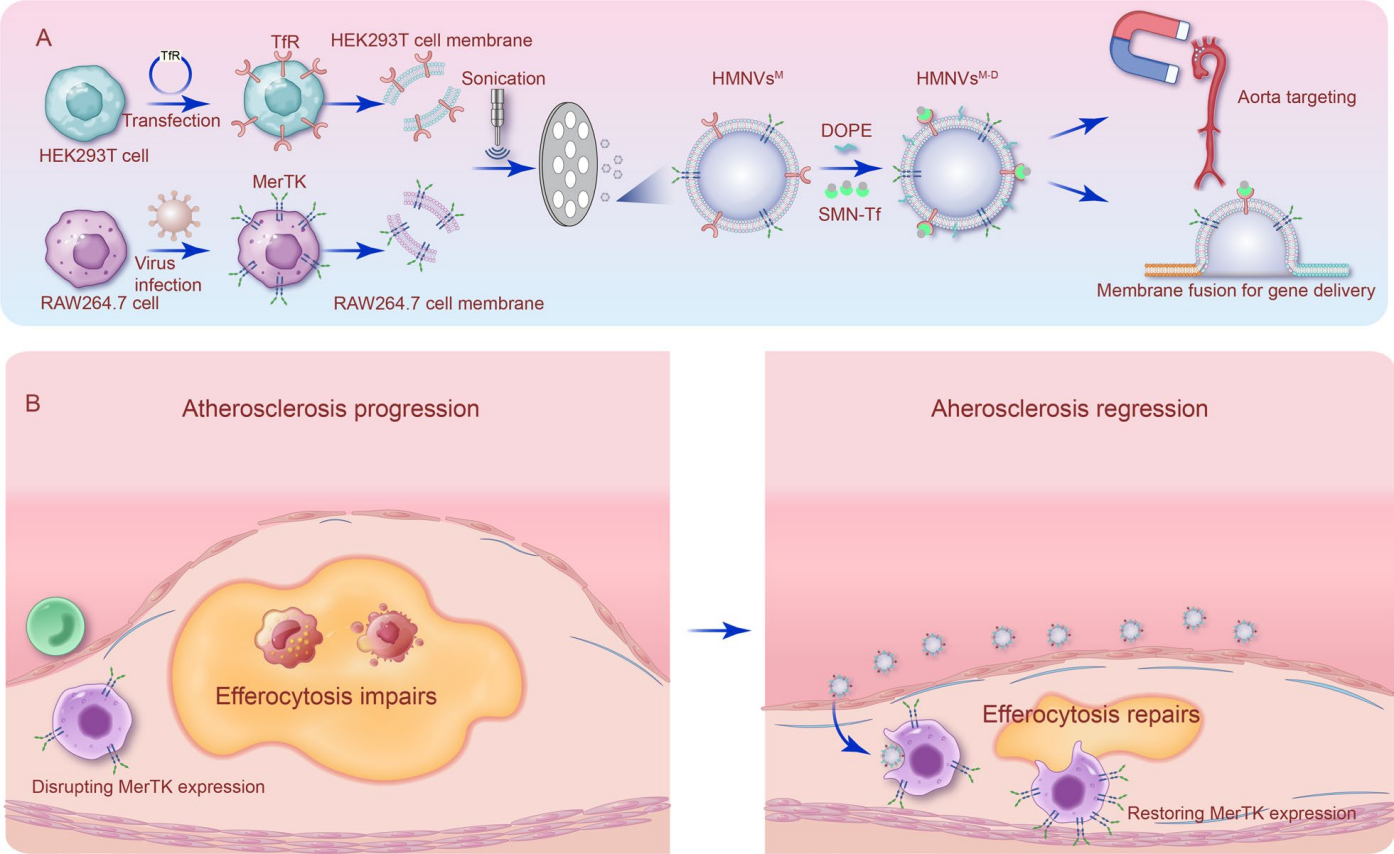

**Supplementary Information:**

The online version contains supplementary material available at 10.1186/s12951-024-02463-y.

## Introduction

Cardiovascular diseases (CVDs), primarily attributed to atherosclerosis (AS), emerge as a significant global health threat [1–3]. Diabetes mellitus, in general, is a well-known risk factor for atherosclerosis and CVD [4–6]. The acceleration of atherosclerosis progression and subsequent plaque rupture in individuals with diabetes has resulted in a significant incidence of CVD [7]. However, the precise mechanism underlying this phenomenon remains to be fully elucidated.

Accumulation of apoptotic cells within advanced atherosclerotic plaques is associated with the emergence of necrotic cores, inflammation, plaque disruption, and atherothrombosis [8]. Clearance of apoptotic cells by phagocytes, namely efferocytosis, not only directly removes the dead cell, but also triggers the release of anti-inflammatory cytokines to suppress atherosclerosis [9]. Vulnerable plaque characterized by necrosis, inflammation, and thinning of the fibrous cap is attributed to compromised efferocytosis [10–12]. Defining the detailed mechanism how efferocytosis is dysregulated in atherosclerosis, especially in the context of diabetes, is greatly needed.

Macrophages employ diverse mechanisms to identify, contact, and internalize/digest apoptotic cells through efferocytosis [13]. Phosphatidylserine (PS) exposed on the surface of apoptotic cells plasma membrane is a key molecule utilized by the macrophages [14]. The recognition of PS is predominantly mediated by surface receptors, including the tyrosine kinase receptor family (TAM), T cell immunoglobulin mucin receptor (TIM), brain-specific angiogenesis inhibitor 1 (BAI1), stabilin 2, and members of the CD300 family [15]. Among the TAM family of receptors, Tyro3, Axl, and Mer tyrosine kinase (MerTK), have been shown to play significant roles in the phagocytic clearance of apoptotic cells [16–18]. It is thus interesting to explore whether these receptors are dysregulated in diabetes and restoration of the expression would be therapeutically beneficial.

Recently, novel cell membrane camouflaged synthetic nanoparticles have been developed to improve biocompatibility, targeting, and prolonged blood circulation [19, 20]. Macrophage membrane modified nanoparticles have been empowered with targeting and homing capacity owned by the macrophages [21, 22]. In addition, 1-2Dioleoyl-sn-glycero-3-phosphoethanolamine (DOPE) was applied to improve gene delivery efficiency via membrane fusion [23]. In addition, superparamagnetic iron oxide nanoparticles (SMN) have also been explored in targeted drug delivery owing to their enhanced targeting capabilities, biodegradability, biological compatibility, and minimal toxicity [24].

In this study, we first explored whether and how efferocytosis is impaired in diabetic atherosclerosis. Then, we developed a type of hybrid membrane nanovesicles (HMNVs) to restore macrophage efferocytosis capacity. Our study not only unravels that diabetes aggravates atherosclerosis at least partially via decreasing MerTK expression and thus efferocytosis in macrophages, but also indicates that the HMNV-based MerTK delivery could be a promising therapeutic approach.

## Results

### Diabetes aggravates atherosclerosis in ApoE^−/−^ mice

To induce diabetic atherosclerosis, streptozotocin was injected into ApoE^−/−^ mice. And wild type C57BL mice served as controls. The mice were fed with a high-fat diet for a duration of 10 weeks (Additional file 1: Figure S1A). No plaques were observed in the wildtype mice. In contrast, ApoE^−/−^ + STZ mice displayed significantly larger plaque lesions, as assessed by Oil red O staining of the artery (Additional file 1: Figure S1B-1D and S1H). Consistently, cross-sectional view of necrotic core abundance in HE staining analysis (Additional file 1: Figure S1E and S1I) and histological analysis of the aortic root (Additional file 1: Figure S1F and S1J) revealed that ApoE^−/−^ + STZ mice had larger lesions than ApoE^−/−^ mice. Furthermore, less aortic lesions’ collagen deposition was observed in ApoE^−/−^ + STZ mice (Additional file 1: Figure S1G and S1K), indicating that the plaques in ApoE^−/−^ + STZ mice were more unstable. Compared with control mice, ApoE^−/−^ + STZ mice exhibited significantly higher blood glucose levels, total cholesterol, triglycerides, and low-density lipoprotein (LDL) levels. Conversely, there were no significant differences in high-density lipoprotein (HDL) levels among the groups (Additional file 1: Figure S2A–S2E).

Arterial stiffness is an independent predictor of coronary heart disease and atherosclerosis as measured by pulse wave velocity (PWV). PWV analysis revealed that ApoE^−/−^ + STZ mice had increased arterial stiffness compared with other control groups (Additional file 1: Figure S2F). Collectively, these findings suggest that diabetes exacerbates atherosclerosis in ApoE^−/−^ mice.

### High glucose damages the efferocytosis capacity

**Fig. 1:**
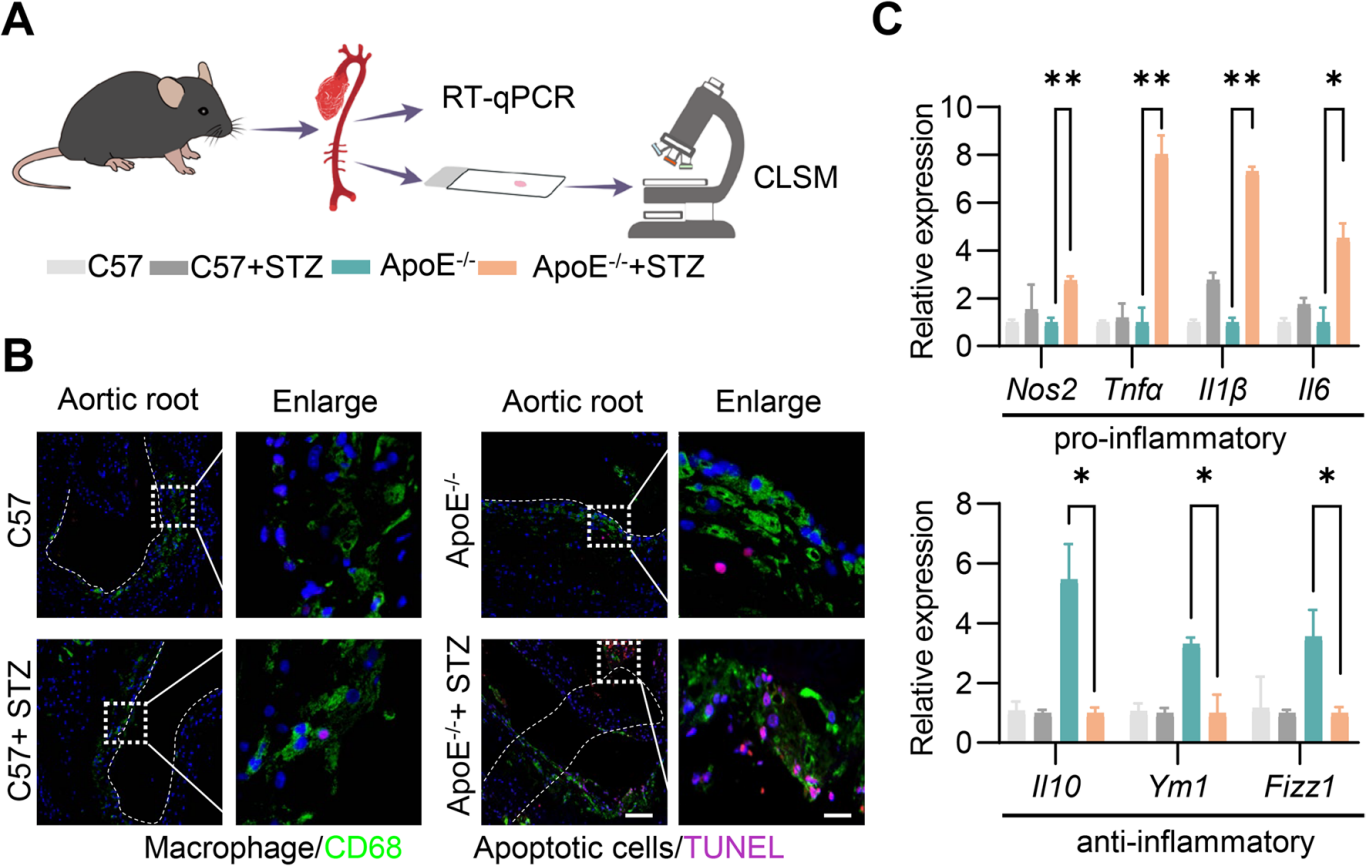
Diabetes aggravates atherosclerotic lesions. **A** Schematic diagram showing animal experiment procedures. Mice were treated as depicted and at the end of the experiment, the heart and aorta were harvested for tissue staining and RT-qPCR analysis. **B** Representative confocal laser scanning microscopy (CLSM) images showing the localization of apoptosis cells by TUNEL staining and CD68^+^ macrophage cells in the atherosclerotic plaques of aortic roots. Scale bar = 100 or 10 µm. **C** RT‐qPCR analysis of pro- and anti-inflammatory cytokine mRNA levels in lesioned aorta. Data are presented as mean ± SEM of three independent experiments. Statistical significance was determined by one-way ANOVA with Tukey’s post hoc test. *P < 0.05 and **P < 0.01

In agreement with the larger plaques, we found that ApoE^−/−^ + STZ mice had increased numbers of terminal deoxynucleotidyl transferase dUTP nick end labeling-positive CD68-positive cells within the plaques (Fig. 1A and B). Accordingly, there was a significant increase in relative gene expression (mRNA) of pro-inflammatory cytokines (*Tnfα, Nos2, Il1β, and Il6*) in ApoE^−/−^ + STZ mice. However, the anti-inflammatory cytokines (*Fizz1, Ym1, and Il10*) exhibited decrease (Fig. 1C). These findings indicated that diabetes mellitus aggravates the progression of atherosclerosis by increasing apoptotic cell and vascular inflammation within the lesions.

To confirm whether these effects are due to high glucose, inflammatory gene expression in bone marrow-derived macrophages (BMDMs) cultured under conditions of high glucose (25 mmol/L) and physiological glucose (5.5 mmol/L), were thus analyzed by RT-qPCR analysis. High glucose promoted the expression of pro-inflammatory M1-related (*Il6, Nos2, and Il1β*) genes and inhibited the expression of anti-inflammatory M2-related (*Fizz1, Ym1, and Il10*) genes (Additional file 1: Figure S3A). Similar results were observed in RAW 264.7 cells (Additional file 1: Figure S3B).

**Fig. 2:**
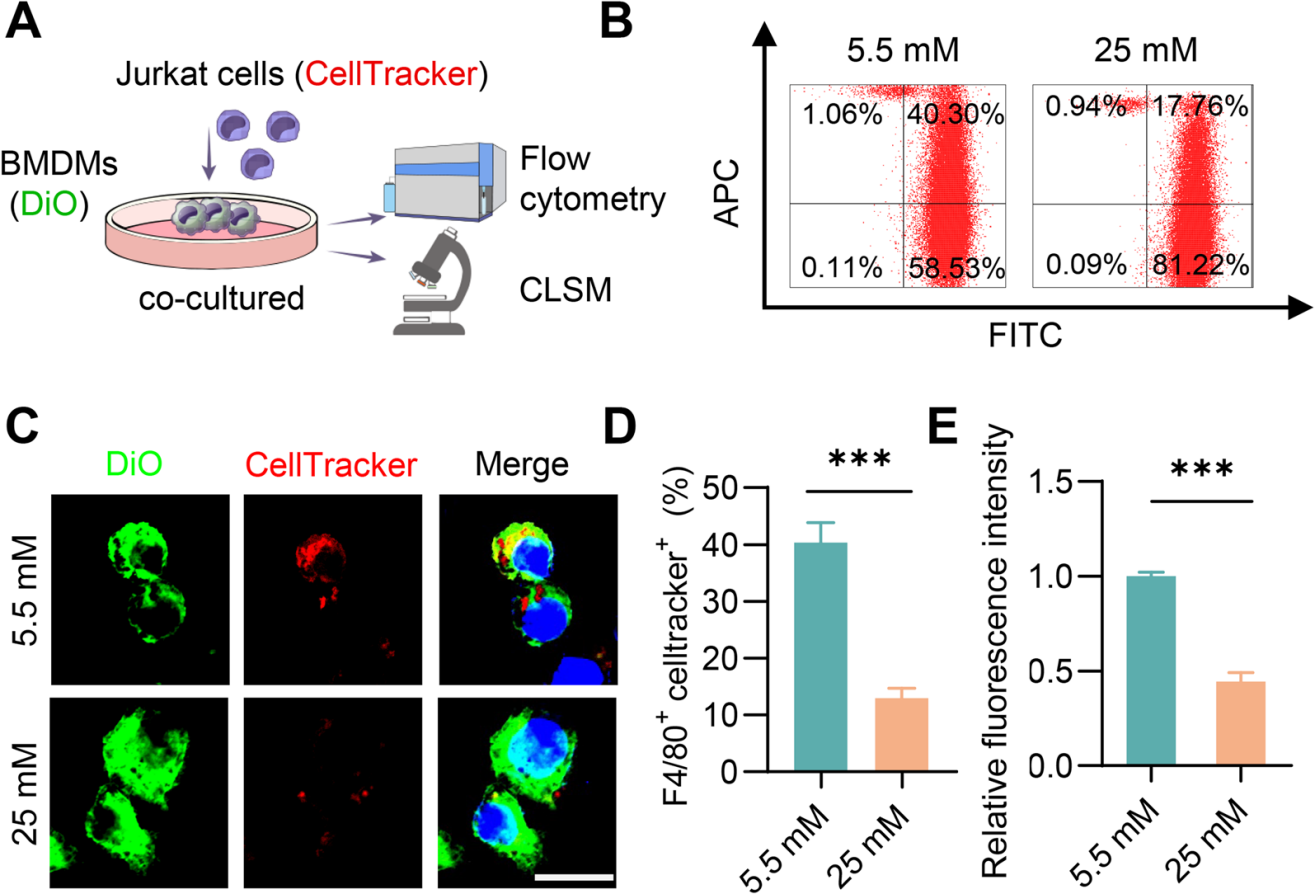
High glucose decreases the efferocytosis capacity. **A** Schematic of Efferocytosis Assay in vitro. DiO labeled macrophages were co-cultured with Celltracker labeled Jurkat cells, followed by flow cytometry and immunofluorescence analysis. **B** Flow cytometry analysis of FITC-F4/80 and Celltracker double positive cells. **C** Representative confocal laser scanning microscopy (CLSM) images showing the localization of apoptotic cells (Jurkat cells) endocytosed by macrophages cells. Scale bar = 10 µm. **D** Quantitative analysis of flow cytometry data. E. Quantitative analysis of data obtained from fluorescence microscopy imaging. Data are presented as mean ± SEM of three independent experiments. Statistical significance was determined by student’s t test, ***P < 0.001

Theoretically, increased apoptosis in the plaque might be also due to the decline in macrophage efferocytosis. To investigate the potential impact of high glucose on macrophage efferocytosis, BMDMs with control or high glucose treatment were incubated with apoptotic Jurkat cells, followed by flow cytometry and confocal fluorescence microscopy (Fig. 2A). Remarkably, high glucose decreased the percentage of double-positive cell (F4/80^+^ and celltracker^+^) in BMDMs, indicating decreased efferocytosis of BMDMs cultured in high glucose by flow cytometry analysis (Fig. 2B and D). Accordingly, similar results were also observed by microscope analysis (Fig. 2C and E). Consistent with the BMDM results, high glucose also decreased the efferocytosis capacity in RAW264.7 cells (Additional file 1: Figure S4A-S4E).

### The reduction of MerTK expression leads to impaired efferocytosis

**Fig. 3:**
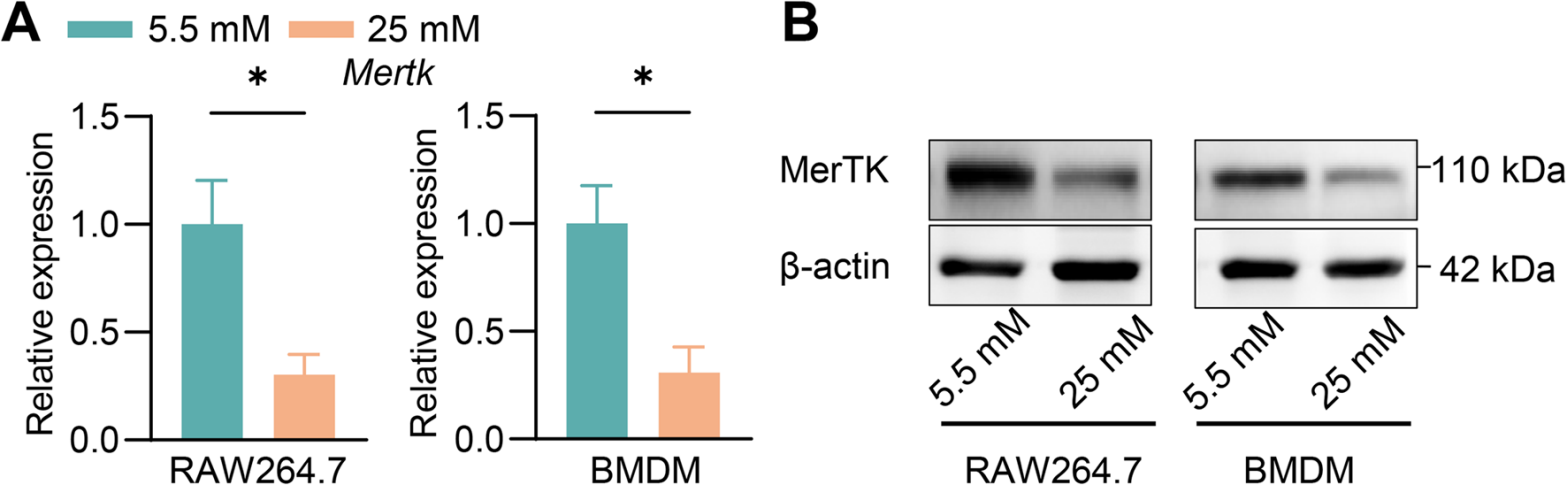
Hyperglycemia impaired efferocytosis while MerTK expression reduction. **A** RT‐qPCR analysis of *Mertk* mRNA levels in RAW 264.7 and BMDMs treated as indicated. **B** Western blot analysis of MerTK expression in RAW 264.7 and BMDMs treated as indicated. Data are presented as mean ± SEM of three independent experiments. Statistical significance was determined by student’s t test, *P < 0.05

The process of efferocytosis is finely regulated by multiple molecules that recognize, engage, engulf, and process the cellular material. Among the gene involved, MerTK is a cell surface receptor and signaling molecule, which plays a crucial role in promoting efferocytosis in macrophages [8]. We found that high glucose levels significantly downregulate *Mertk* mRNA expression (Fig. 3A). Consistently, western blot assays demonstrated that elevated extracellular glucose levels significantly reduced the protein expression of MerTK in both cell lines (Fig. 3B). The results suggest that high glucose may damage efferocytosis capacity by suppressing MerTK expression.

### Hybrid membrane nanovesicles restore efferocytosis capacity in vitro

**Fig. 4:**
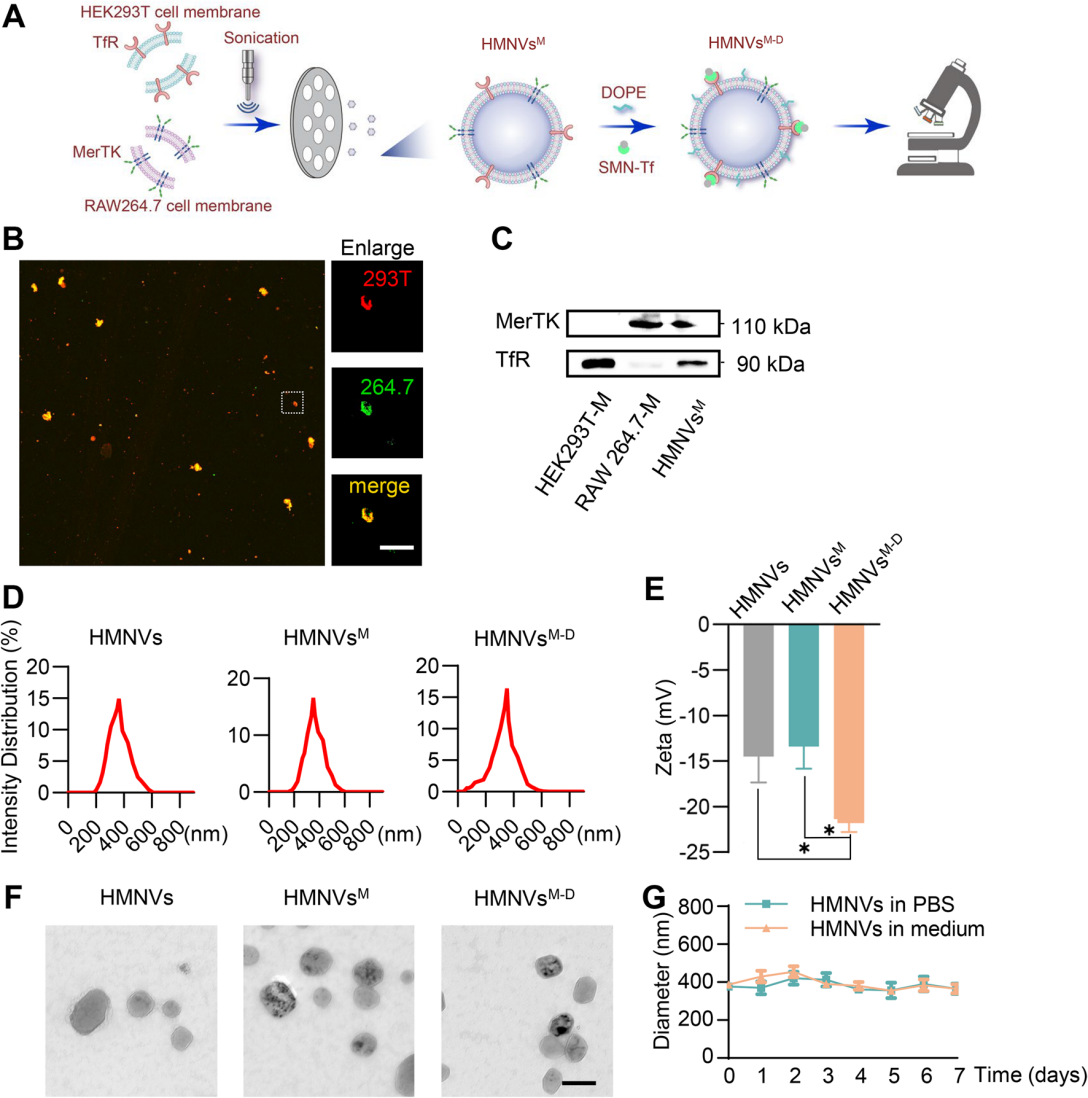
Fabrication and characterization of HMNVs. **A** Schematic illustration for the preparation of HMNVs^M−D^ through the hybridization of cell membrane harvested from RAW264.7 cell-HEK293T cell. **B** Confocal laser scanning microscopy (CLSM) images of fabricated HMNVs. RAW264.7 cell membranes were labeled with DiO (green) and HEK293T membranes were labeled with DiI (red). Scale bar = 500 µm. **C** Western blot analysis of MerTK and TfR expression in HMNVs. **D** Size distribution of HMNVs, HMNVs^M^, HMNVs^M−D^. **E** Zeta potential of HMNVs, HMNVs^M^, HMNVs^M−D^. **F** Representative TEM images of HMNVs, HMNVs^M^, HMNVs^M−D^. Scale bar = 500 nm. **G** Particle size change of HMNVs^M−D^ in PBS and medium. Data are presented as mean ± SEM of three independent experiments. Statistical significance was determined by one-way ANOVA with Tukey’s post hoc test. *P < 0.05

The above data showed that MerTK may represent a crucial target for therapeutic intervention in the context of diabetic atherosclerosis. To restore macrophage efferocytosis via targeted MerTK delivery, a hybrid membrane nanoparticle was then developed (Fig. 4A). First, *Mertk* gene overexpression on RAW264.7 cells was achieved by lentiviruses infection, and high expression level was confirmed by Western Blot (Additional file 1: Figure S5). Similarly, transferrin receptor (TfR) gene overexpression on HEK293T cell was achieved by plasmid transfection. RAW264.7 cells membranes and HEK293T cell membranes were prepared by the ultrasonic approach and coextruded to form a hybrid cell membrane vesicle, successful hybrid was demonstrated by confocal microscopy (Fig. 4B). Finally, SMN (10 nm, Nanoeast, China) and DOPE were co-incubated with HMNVs to get HMNVs^M−D^. SMN shows good dispersion (Additional file 1: Figure S6A) and can be aggregated under magnetic field (Additional file 1: Figure S6B). As shown in Fig. 4C, the TfR and MerTK proteins were observed on HMNVs. Nanoparticle tracking analysis (NTA) showed that HMNVs, HMNVs^M^ and HMNVs^M−D^ had similar size distributions, with diameters ranging from 300 to 400 nm (Fig. 4D). In addition, surface charge changed from − 10–15 mV to − 20–25 mV after the membranes were connected with DOPE (Fig. 4E). The nanoparticle structure of the HMNVs, HMNVs^M^ and HMNVs^M−D^ was confirmed with the transmission electron microscopy (TEM) (Fig. 4F). Notably, HMNVs^M−D^ maintained size stability for at least 7 days in PBS solution and DMEM medium (Fig. 4G).

**Fig. 5:**
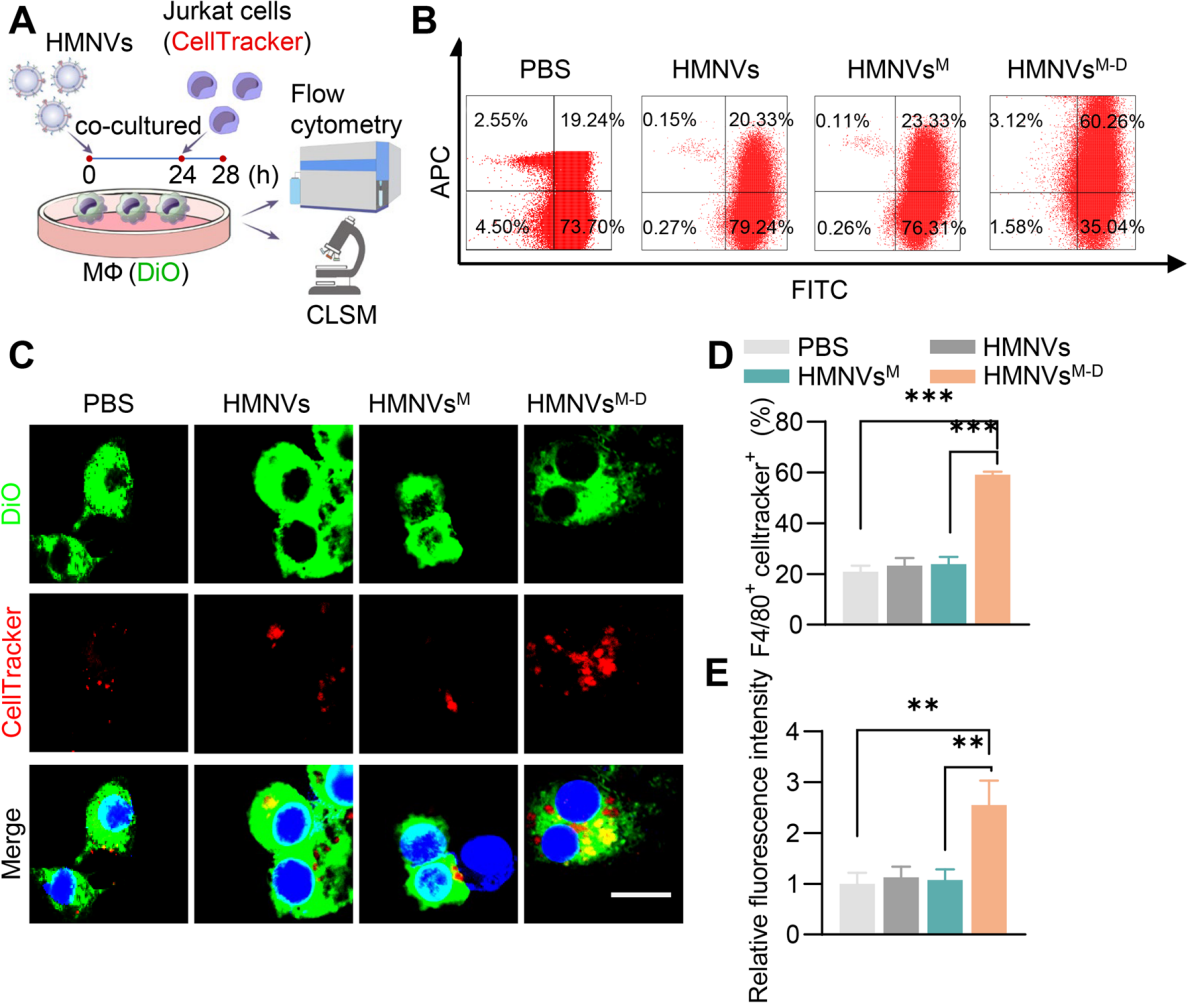
HMNVs restores efferocytosis capacity in vitro. **A** Schematic of efferocytosis assay in vitro. DiO labeled macrophages were co-cultured with Celltracker labeled Jurkat cells and then underwent immunofluorescence and flow cytometry analysis. **B** Efferocytosis capacity were detected with flow cytometry. **C** Representative confocal laser scanning microscopy (CLSM) images showed the localization of apoptotic cells (Jurkat cells) endocytosed by macrophages. Scale bar = 10 µm. **D** Quantitative analysis of flow cytometry data. **E** Quantitative analysis of the fluorescence data. Data are presented as mean ± SEM of three independent experiments. Statistical significance was determined by one-way ANOVA with Tukey’s post hoc test. **P < 0.01, ***P < 0.001

In the following experiments, we explored whether HMNVs^M−D^ restore macrophage efferocytosis in vitro (Fig. 5A). HMNVs, HMNVs^M^ and HMNVs^M−D^ had no obvious effects on cell viability (Additional file 1: Figure S7). Confocal fluorescence microscopy and flow cytometry analysis revealed an enhancement in the efferocytosis function of the HMNVs^M−D^ (Fig. 5B–E).

### Therapeutic effects of HMNVs in attenuating atherosclerosis in diabetic ApoE^−/−^ mice

**Fig. 6:**
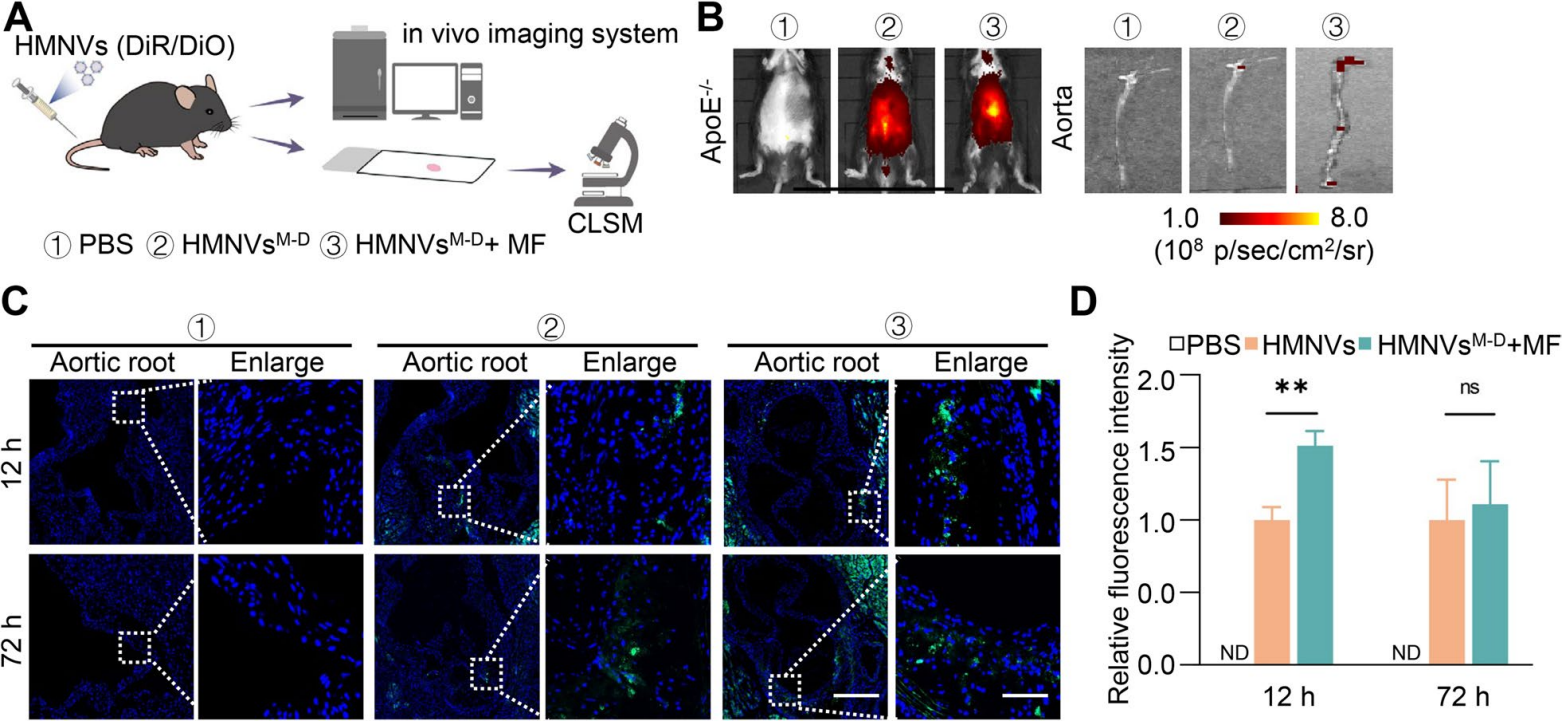
Biodistribution of HMNVs in ApoE^−/−^ mouse. **A** Schematic of biodistribution of HMNVs. HMNVs (labeled and unlabeled with DiO/DiR) were injected into ApoE^−/−^ mice with or without MF, then analyzed by live imaging and immunofluorescence for tracking at 12 h or 72 h post-injection. **B** Representative in vivo imaging system (IVIS) images of mice and aorta injected with HMNVs via tail vein. **C** Representative confocal laser scanning microscopy (CLSM) images of the HMNVs in the aorta root (green). The nuclei were counter-stained with Hoechst (blue). Scale bar = 500 or 100 μm. **D** Quantitative analysis of the fluorescence intensity. Data are presented as mean ± SEM (n = 3 per group). Statistical significance was determined by one-way ANOVA with Tukey’s post hoc test. **P < 0.01. ns, no significance

Before exploring the effects of HMNVs in the treatment of diabetic atherosclerosis, we first analyzed biodistribution of HMNVs^M−D^ in ApoE^−/−^ mice after systemic administration. HMNVs^M−D^ were labeled with DiO/DiR and injected into ApoE^−/−^ mice via the tail vein and MF was applied to the aorta area for one hour in the MF group (Fig. 6A). HMNVs^M−D^ were found distributed to liver and spleen, as revealed by live imaging analysis (Additional file 1: Figure S8A–S8E). Notably, an increased quantity of HMNVs^M−D^ was present in the aorta by magnetic field (MF) (Fig. 6B–D), and the effects could be last for at least 72 h. The above results suggest with the long-term stability and circulating time of the HMNVs^M−D^, which is consistent with previous studies [25, 26].

**Fig. 7:**
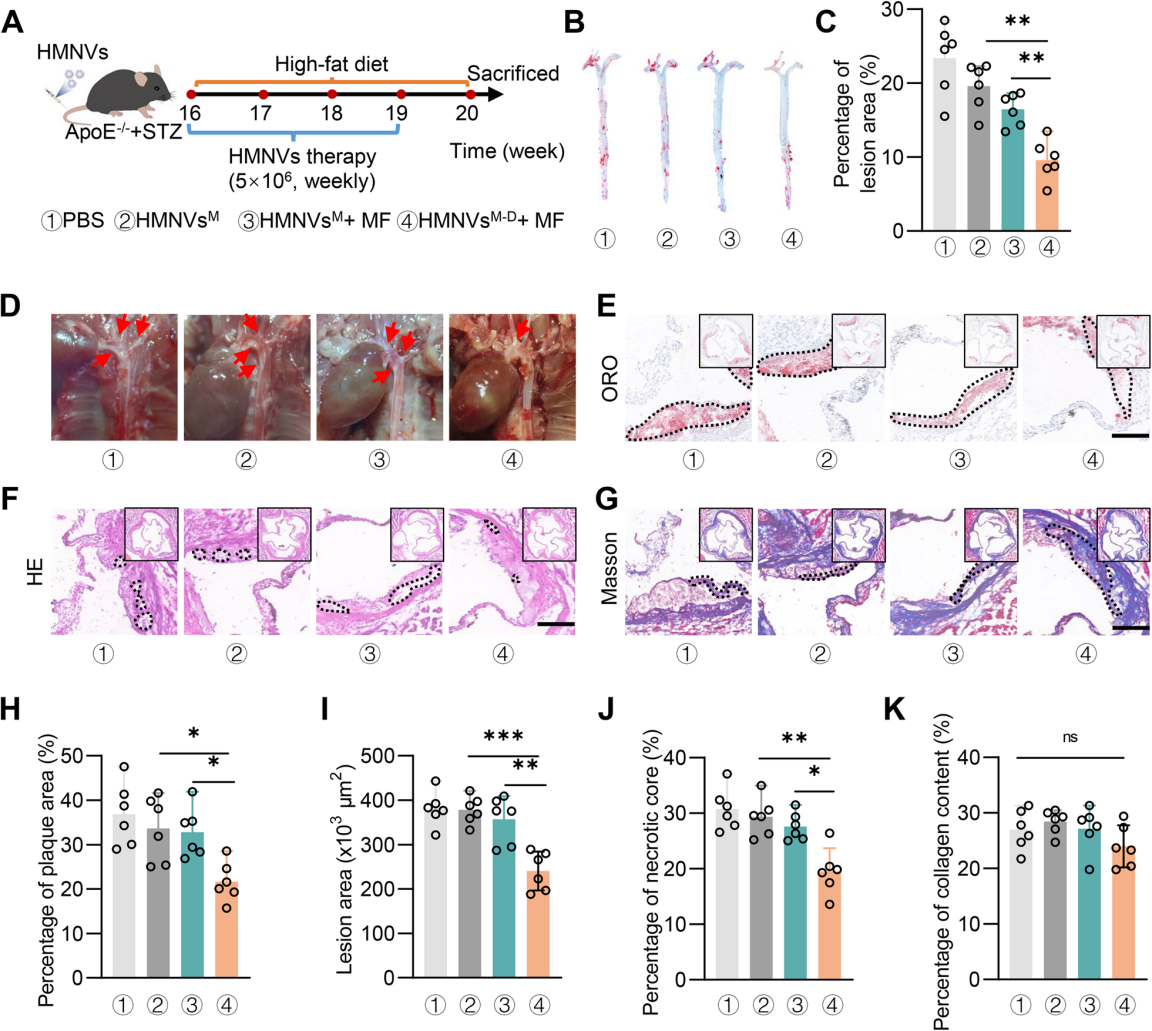
HMNVs^M−D^ together with MF attenuate atherosclerotic lesions in ApoE^−/−^ mice. A. Schematic of the experimental procedure. PBS, HMNVs^M^, HMNVs^M−D^ were injected into ApoE^−/−^ mice via the tail vein weekly. **B** Representative images of Oil Red O staining of the atherogenic lesion area in mice. **C** Percentage of the aortic lesion area. **D** Representative aortic arch view of the atherosclerotic lesions in ApoE^−/−^ mice treated as indicated. **E**–**G** Representative images of the atherogenic lesion area stained with hematoxylin and eosin (H&E), Masson’s trichrome, and Oil Red O. Scale bar = 200 μm. **H**–**K** Quantitative analysis of the aortic lesion, necrotic core area, plaque collagen area relative to plaque area, lesion areas relative to plaque area. All data are expressed as mean ± SD (n = 6 per group). Statistical significance was determined by one-way ANOVA with Tukey’s post hoc test. *P < 0.05, **P < 0.01, ***P < 0.001. ns, no significance

Next, ApoE^−/−^ mice were administered with HMNVs^M−D^ via tail vein once a week while a MF was applied to the aorta for one hour (Fig. 7A). As expected, HMNVs^M−D^ + MF treatment significantly decreased plaque development in ApoE^−/−^, as assessed by Oil red O staining of the artery, histological analysis of the aortic root, plaque necrotic core abundance in histological cross sections of the aorta, and collagen deposition of aortic lesions by Masson's trichrome staining (Fig. 7B–K). Notably, no significant change on the collagen deposition was found, suggesting the therapy has no effects on the plaque stability. Blood glucose and lipid profiles in HMNVs^M−D^ treatment showed no significant difference from those of PBS treatment and HMNVs^M^ treatment (Additional file 1: Figure S9A–S9E), suggesting that the therapeutic effects were independent of lipid levels. Notably, PWV in HMNVs^M−D^ + MF treatment didn’t change (Additional file 1: Figure S9F). In conclusion, these findings suggest that the HMNVs^M−D^ attenuates atherosclerosis in diabetic ApoE^−/−^ mice in a metabolism independent manner.

### Safety of HMNVs^M−D^ treatment

**Fig. 8:**
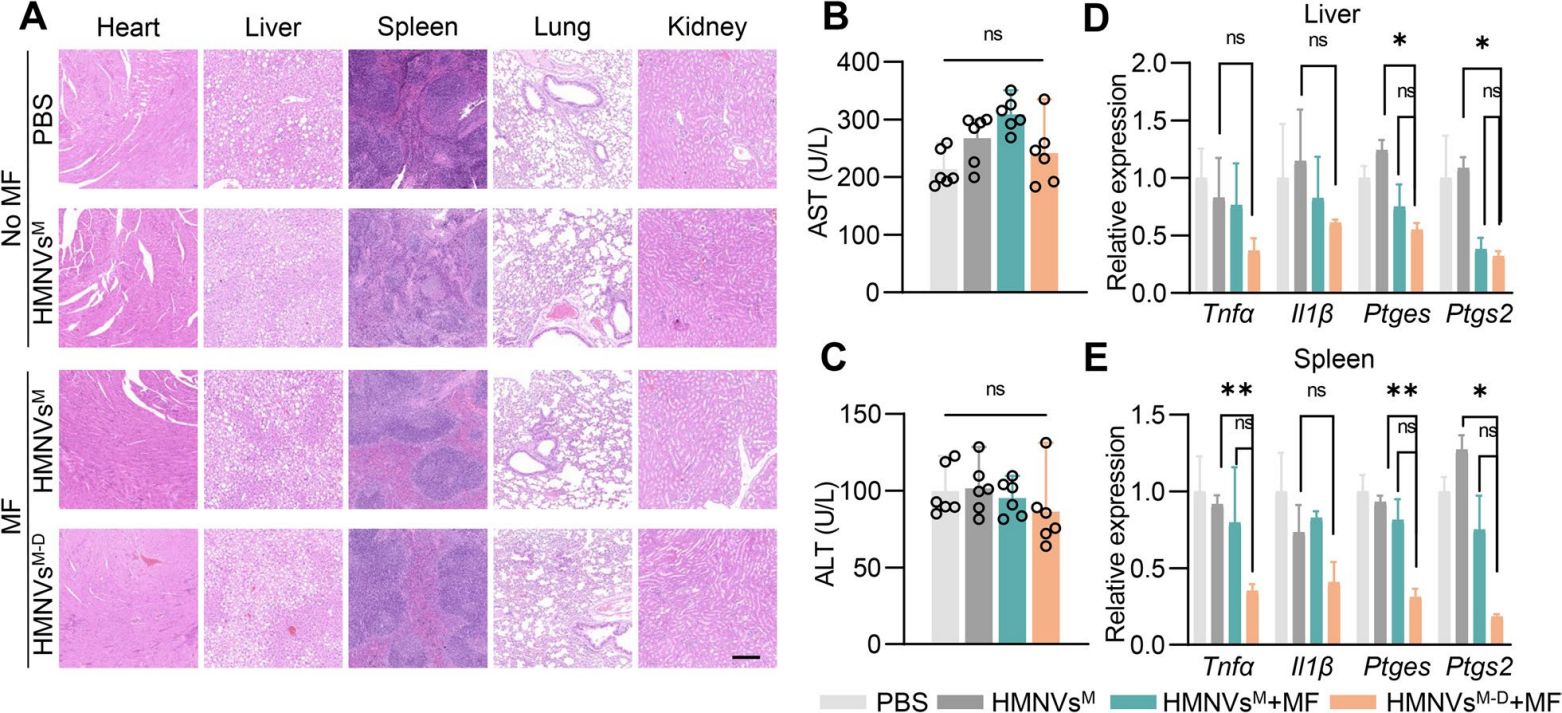
Safety of HMNVs^M−D^ therapy. **A** H&E staining of different organs from mice of indicated groups. Scale bar = 500 μm. **B**–**C** Examination of the ALT and AST in mice treated as indicated. **D**–**E**. RT‐qPCR analysis of *Tnfα*, *Il1β, Ptges and Ptgs2* mRNA levels in the liver or spleen of mice with indicated treatments. Data are presented as mean ± SEM of three independent experiments. Statistical significance was determined by one-way ANOVA with Tukey’s post hoc test. *P < 0.05, **P < 0.01. ns, no significance

In the following experiments, we investigated the possible side-effects of HMNVs^M−D^ in vivo. HE staining analysis of the main organs revealed that there was no apparent toxicity (Fig. 8A). HMNVs^M−D^ + MF treatment had a negligible impact on aspartate aminotransferase (AST) and alanine transaminase (ALT) (Fig. 8B and C). Additionally, qPCR assays demonstrated a significant reduction in the expression of inflammatory genes (*Il1β*, *Tnfα*, *Ptges*, *Ptgs2*) following HMNVs^M−D^ + MF treatment (Fig. 8D and E), suggesting that restoration of efferocytosis in liver could be also beneficial in liver. Cardiac function analyzed echocardiography revealed that HMNVs^M−D^ + MF treatment had no discernible harm to cardiac structure or function (Additional file 1: Figure S10A–S10D). Taken together, these findings provide further evidence that HMNVs^M−D^ treatment did not elicit any observable toxic effects in the heart, liver or spleen.

## Discussion

The study revealed that diabetes exacerbates the progression of atherosclerosis in ApoE^−/−^ mice, at least partially due to damaged efferocytosis capacity. Mechanistically, hyperglycemia decreased the expression of MerTK on macrophages. Delivery of MerTK via magnetic navigated HMNVs restored MerTK in diabetic macrophages and thus rescued the efferocytosis capacity, serving as a promising therapeutic approach to alleviate atherosclerosis.

Studies have consistently demonstrated that diabetic patients exhibit a greater incidence of cardiovascular morbidity and mortality when compared to the general population [27–29]. There are several factors associated with diabetes that exacerbated the progression of atherosclerosis, including elevated levels of atherogenic LDL, hyperglycemia, oxidative stress, and heightened inflammation [30]. Hyperglycemia in diabetes causes endothelial dysfunction and structural damage [31, 32]. However, conventional glycemic intervention strategies are ineffective in treating atherosclerosis, which is in contrast to their effectiveness in treating microvascular lesions [33]. Notably, the pathogenesis of atherosclerosis is intricately linked to inflammation, dyslipidemia, and other risk factors. For example, SGLT2 inhibitors and GLP1 receptor agonists are found to reduce diabetes-related macrovascular complications via mechanisms beyond glucose reduction [34–36]. Efferocytosis is a crucial process in maintaining tissue homeostasis [37]. This biological phenomenon refers to the efficient clearance of programmed dead cells, primarily by macrophages and dendritic cells. However, in certain chronic inflammatory conditions, such as atherosclerosis, efferocytosis may become impaired, leading to the accumulation of dead cells [15]. Impaired efferocytosis in macrophages plays a significant role in the development and advancement of unstable plaques due to apoptotic cell accumulation and secondary necrosis [11, 38]. Studies have demonstrated that the deliberate restoration of macrophages efferocytosis in vivo can effectively and promptly eliminate apoptotic cells (ACs) that accumulated at the lesion site [39]. Efferocytosis is inextricably linked to inflammatory feedback, lipid deposition, and cellular aging, which are important pathophysiological changes caused by diabetes mellitus [16, 40]. Our further study here reveals that diabetes exacerbates atherosclerosis at least partially by damaging macrophage efferocytosis capacity via decreased MerTK. Restoration of macrophage efferocytosis function may serve as a potential therapeutic approach for diabetes-induced atherosclerosis. Theoretically, if MerTK permanently decreased by hyperglycemia via epigenetic mechanisms, conventional glycemic intervention strategies would fail to restore MerTK, which is worth to be explored. It is also important to note that whether excessive/continuous supplement of MerTK is beneficial for atherosclerosis treatment even when blood glucose is controlled. More MerTK will enhance the efferocytosis and thus be beneficial in the context of atherosclerosis, either with or without diabetes.

Efferocytosis is executed by specific proteins expressed in the macrophage and fine-tuned by many signal pathway [15]. It has been demonstrated that MerTK in macrophages plays a crucial role in the formation of necrotic atherosclerotic plaque, and cleavage of the MerTK leads to plaque necrosis [16, 38]. Monocyte-derived ROS could activate ADAM17 on resident macrophages and facilitate the cleavage of MerTK [41, 42]. In addition, glycolytic reprogramming in macrophages due to hypoxia also results in the cleavage of MerTK and subsequently impaired efferocytosis [43]. Ox-LDL also can promote MerTK cleavage [44]. Besides cleavage, studies have confirmed that CaMKII (Ca^2+^/calmodulin-dependent protein kinase II) and ILRUN (inflammation and lipid regulator with UBA-like and NBR1-like domain) reduce the expression of MerTK [38, 45]. CaMKIIγ siRNA delivery with nanoparticles could increase MerTK expression in the plaque [39]. Our investigation revealed that elevated glucose level decreased the expression of MerTK at both RNA and protein levels.

In exploration the strategy to restore MerTK expression and thus efferocytosis, we here developed HMNV-based platform. Cell membrane-coated nanoparticles have the features of tissue targeting, stealthy and bio-friendly. [19, 20] The membrane can be derived from various cell types [46–48], which inherit the biological properties of the source cells [49]. For various purposes, a combination of cell membranes from different cell sources could be used. For instance, RBC–platelet hybrid membrane-coated nanoparticles were developed to manage atherosclerotic progression [19]. In this study, the HMNVs were composed of MerTK overexpressing RAW264.7 and TfR overexpressing HEK293T. In the HMNVs, MerTK served as the drug and TfR for SMN loading onto the surface for magnetic navigated delivery [24, 50]. In addition, the RAW264.7 cell membrane also makes membrane fusion with endogenous macrophages easier. To further improve membrane fusion, DOPE was added [23, 51]. Through these modifications, the HMNVs^M−D^ are empowered with higher targeting and more efficient plasma membrane fusion capacity, towards better MerTK functional delivery.

Unlike common drug loading strategies, the MerTK protein was loaded onto the HMNVs surface by gene manipulation in present study. Future studies are needed to develop specific and practical method and standard to calculate the loading efficiency. Moreover, HMNVs^M−D^ might interact with the plasma proteins and thus change their in vivo distribution and stability [52]. Specifically, there are SMNs on the surface even though the HMNVs are mainly made of cell membranes. Decoration with the plasma proteins might also change the endocytosis capacity [22]. Future work should explore how plasma proteins change the stability, biodistribution and function of HMNVs^M−D^.

## Conclusions

Our investigation indicates that decrease of MerTK plays a crucial role in atherosclerosis in diabetic mice, and restoration of MerTK could mitigate atheroslcerosis potentially through promoting efferocytosis. Hybrid membrane nanovesicle-based MerTK protein delivery presents as a promising therapeutic approach for atherosclerosis management in patients with diabetes.

## Materials and methods

### Animal experiments

All animal experimental procedures were approved by the Institutional Animal Care and Use Committee at Air Force Medical University. Male ApoE^–/–^ mice (C57/BL background) were purchased from the Model Animal Research Center of Nanjing University. ApoE^–/–^ mice and C57BL mice, aged 6 weeks, were randomly assigned to control or diabetic experimental groups. Then diabetic experimental groups were induced by intraperitoneal injection of STZ (50 mg/kg, Sigma) over five consecutive days. All groups received a high-fat diet for 10 weeks, followed by a blood glucose test. Diabetic group was confirmed by nonfasted blood glucose levels > 13.9 mmol/L.

### Serum biochemistry

Prior to blood sample collection, the mice were fasted for 8 h. The Chemray 800 at Wuhan Servicebio Technology Co., Ltd. was utilized to perform analyses on blood lipid levels and liver function parameters.

### Histology

Following euthanasia, the mice underwent perfusion with 3–5 mL of PBS to facilitate organ dissection. The aortic arch bifurcation was imaged using a stereomicroscope equipped with a digital camera. The heart and aorta were then extracted and cleared of adjacent fat and connective tissues. To quantify plaque burden, paraformaldehyde-fixed aortic roots were placed in optimal cutting medium and sectioned on a plane parallel to the atria. To quantify plaque volume, plaque lipid content, and collagen content, 5 sections, evenly distributed through the aortic root, were H&E, Oil-red-O, and Masson's trichrome stained. ImageJ was utilized to calculate the dimensions of the lesions and lipid cores.

For detection of apoptotic cells in aortic region, aorta sections were fixed and subjected to staining with CD68 (ab197519, Abcam), TUNEL (Sigma Aldrich) and Hoechst (Invitrogen). The entire process was conducted in dark. The fluorescence signals were observed using a laser-scanning confocal fluorescence microscope (A1R, Nikon, Tokyo, Japan).

### Cell culture

RAW 264.7 cells were cultured in high-glucose or low-glucose Dulbecco's modified Eagle's medium (Gibco, Carlsbad, USA) supplemented with 10% fetal bovine serum (FBS, Logan, USA), 1% penicillin/streptomycin solution (Solarbio, China). Jurkat cells were cultured in Roswell Park Memorial Institute 1640 medium (RMPI 1640, Gibco, Carlsbad, USA) supplemented with 10% fetal bovine serum (FBS, Logan, USA), 1% penicillin/streptomycin solution (Solarbio, China). The cell cultures were maintained at 37 °C with 5% CO_2_ humidified atmospheres. Every 1–2 days, culture medium was replaced with fresh medium.

### BMDMs

BMDMs used in this study were generated by flushing murine femurs and tibias and plating on petri dishes in complete DMEM (supplemented with 10% FBS, 2% penicillin–streptomycin, and 2 mmol/L glutamine) plus 20 ng/ml macrophage colony-stimulating factor (M-CSF, Peprotech, USA) for differentiation. BMDMs were simultaneously cultured in various glycemic conditions (25 mmol/L, 5.5 mmol/L glucose DMEM) or an osmotic control condition (mannitol; 5.5 mmol/L glucose DMEM plus 15 mmol/L mannitol) supplemented with 20 ng/ml M-CSF for a duration of 7 days.

### RNA isolation and real-time polymerase chain reaction

The isolation of total RNA from cells and tissues was carried out using Trizol reagent (Invitrogen) in accordance with the manufacturer's instructions. Reverse transcription was performed using PrimeScript First-Strand cDNA Synthesis Kit (Roche), followed by qPCR reactions using FastStart Essential DNA Green Master (Roche) on a LightCycler 96 (Roche). Each PCR reaction was conducted at least three times. The data were standardized to β-actin expression, and the relative gene expression was determined using the 2^−ΔΔCt^ method. The PCR primer sequences are presented in Additional file 1: Table S1.

### Efferocytosis assay

BMDMs and RAW 264.7 cells were cultured in high or low glucose DMEM (25 mmol/L or 5.5 mmol/L) and seeded in confocal dishes. The cells were then labeled with DiO at 37°C for 10 min and washed three with PBS. Jurkat cells were stained with CellTracker Deep Red dye (C34565, Invitrogen, USA) at a dilution of 1:1000. To induce apoptosis, Jurkat cells were exposed to UV radiation for 20 min and subsequently cultured under normal conditions for 3 h. Apoptotic Jurkat cells were introduced into macrophages at a ratio of 5:1 for a duration of 90 min. The unbound apoptotic cells were cleared, and the attached cells were fixed using 4% paraformaldehyde for subsequent confocal microscopy analysis or collected for flow cytometry. Efferocytotic macrophages were characterized as cells that exhibiting Deep Red and F4/80 markers. The efferocytotic rate was determined by calculating the ratio of double-positive cells to all F4/80 positive cells.

### Western blot analysis

Protein of each sample was prepared using RIPA lysis buffer (Beyotime, China), and quantified using the Pierce BCA Protein Assay Kit (Thermo Fisher Scientific, USA). The samples were separated by electrophoresis by Sodium Dodecyl Sulfate Poly-Acrylamide Gel Electrophoresis (SDS-PAGE) in 6–10% gels, and transferred onto nitrocellulose filter membranes (Millipore). The membrane was blocked with 5% bovine serum albumin (BSA) in Tris-buffered saline with 0.1% Tween 20 (TBS-T) for 1h at room temperature and incubated overnight at 4℃ with each primary antibody. Primary antibodies used included anti-MerTK polyclonal antibody (ab300136, Abcam) anti-transferrin receptor (TfR) polyclonal antibody (10,084–2-AP, Proteintech), anti-β-actin polyclonal antibody (81115-1-RR, Proteintech). Membranes were subsequently washed three times with TBST for 5 min each and incubated with anti-rabbit or anti-mouse horseradish peroxidase-conjugated secondary antibodies for 1 h at room temperature. The specific protein bands were visualized using an enhanced chemiluminescence (ECL) western blotting substrate (34,580, Thermo Fisher Scientific, USA).

### Virus infection in macrophage

To perform lentivirus infection in vitro, BMDMs were seeded into 6-well plates and subjected to the aforementioned conditions. The cells were then infected with lentiviruses expressing control or *Mertk*, in the presence of 8 mg/ml polybrene (Sigma, St. Louis, USA). After 12 h, the infected cells were switched to fresh medium containing 10% FBS and cultured for an additional 48 h.

### Preparation of hybrid membranes nanovesicles (HMNVs)

Cell membrane fragments collected from RAW 264.7 cells and 293T cells were obtained according to the manufacturer’s instructions. Briefly, cells were scraped from cell culture plates with phosphate-buffered saline (PBS) and washed with PBS three times by centrifugation at 200×*g*. The cells were suspended in hypotonic lysis buffer containing Complete Tablets mini protease inhibitor (Roche, Switzerland), and incubated in an ice bath for 30 min. The cells were subsequently disrupted via homogenization with Dounce homogenizer (Sigma, USA). The cell homogenate was then centrifuged at 700 g (10 min, 4 °C) to remove nuclei and unbroken cells, after which the supernatant was centrifuged again at 10,000 g (10 min, 4 °C) to remove mitochondria and other organelles. The resulting supernatant was centrifugated at 100,000 g (60 min, 4 °C), and the pellets were washed three times in PBS. The resulting membrane pellets were stored at − 80 °C [53].

### Fabrication of hybrid membranes nanovesicles (HMNVs)

HMNVs was fabricated using the previously reported method. [54] RAW 264.7 cells with MerTK overexpression and 293 T cells with TfR overexpression were mixed at the ratio of 1:1 (mass ratio of protein detected by BCA protein assay kit) and sonicated for 10 min in an ice-water bath, which does not cause protein damage and is routinely used to extract membrane protein [48, 56, 57]. Then membranes were physically extruded through a 800 nm polycarbonate membrane for 15 passes. The mixture membrane solution was subsequently extruded through a 400 nm polycarbonate membrane for 15 passes. The final solution was storage at 4 °C for further use.

For visualization of membrane hybridization under confocal laser scanning microscopy analysis, DiO-labeled RAW 264.7 cells membrane vesicles and DiI-labeled 293T cells membrane were subjected to freeze–thaw process before extrusion. The colocalization of DiO and DiI signal was observed under confocal laser scanning microscopy.

To fabricate HMNVs^M^, SMN-Tf was first synthesized using the previously reported method [57]. Briefly, a solution of SMN (20 μL, 4 mg/mL, Nanjing, China) was mixed with EDAC and sulfo-NHS (sigma, St Louis, MO, USA) in a molar ratio of 1:2:3 (pH = 5.5). The resultant mixture was incubated at ambient temperature for 1 h, followed by the addition of 1 μL of 2-mercaptoethanol to halt the reaction. The activated SMN were separated magnetically and subsequently resuspended in 200 μL of borate buffer (20 mM, pH = 8.5). Subsequently, 10 μg of Tf was introduced to the solution, and the mixture was subjected to a 12-h incubation at 4 °C under nitrogen. Following magnetic separation, SMN-Tf was isolated, washed three times with PBS, and resuspended in 200 μL PBS. It was then stored at 4 °C for subsequent experimental procedures. Then, 100 μL HMNVs with TfR displayed on the surface was mixed with 100 μL of SMN-Tf solution and incubated for 4 h at 4 °C on a shaker. Subsequently, the HMNVs^M^ was obtained through magnetic separation and resuspended in PBS for subsequent experiments.

To fabricate HMNVs^M−D^, HMNVs^M^ were resuspended with DOPE solution (1 mg DOPE powder dissolved in 1 mL PBS) and incubated at 4 °C overnight. The decorated HMNVs were isolated and termed as HMNVs^M−D^.

The morphology of HMNVs were determined using TEM (JEM1400, JEOL, Japan). The size distribution of HMNVs was analyzed via NTA using Nanosight (NS300, Malvern, UK). Moreover, the ζ-potential of these nanoparticles were tested with a Zetasizer (Nano ZS, Malvern, UK).

### Cell uptake and cytotoxicity

For cell uptake assay, BMDMs (1 × 10^4^ cell per well) were seeded in confocal dishes. After 24 h, the cells were incubated with HMNVs, HMNVs ^M^, HMNVs^M−D^ for 4 h. Then, apoptotic Jurkat cells were added and efferocytosis was analyzed as previous described. And cell uptake indicated with HMNVs fluorescence was observed using the aforementioned protocol. Cytotoxicity assay was measured using a CCK-8 kit (Yeasen, 40203ES60). RAW 264.7 cells by seeded in 96-well plates and co-incubated with HMNVs at a final concentration of 100 µg/mL at 80% cell confluency. After 24 h, the CCK-8 solution was added, and absorbance was measured at 450 nm using a microplate reader (EPOCH, Bio-Tek) to determine cell viability.

### Biodistribution

For in-vivo biodistribution and aorta targeting efficiency, HMNVs, HMNVs^M^, HMNVs^M−D^ were labeled with DiO or DiR at 37 °C for 10 min and subsequently injected into ApoE^–/–^ + STZ mice via tail vein. Magnetic field was performed in indicated group. After 12 or 72 h, all mice were anesthetized with 1% pentobarbital sodium (50 mg/kg) and the main organs were harvested and fixed with 4% paraformaldehyde (PFA; Thermo Fisher). In vivo and ex vivo distribution of HMNVs was assessed using the IVIS® Lumina II system (PerkinElmer, Waltham, USA) in accordance with the provided instructions. Confocal fluorescence microscopy was used to monitor DiO labeled HMNVs in different tissues.

### Echocardiography

The mice were prepared with the anterior chest hair removed using a chemical hair remover before subjected to anesthesia with isoflurane (2% induction, 1.2% maintenance). Then, the mice were placed on a temperature-controlled heating pad to ensure normothermia. To assess cardiac contraction, diastolic function, and PWV, echocardiography was conducted on the mice by skilled technicians using a Vevo 2100 Imaging System (FUJIFILM VisualSonics, Toronto, Canada). During the examination, the heart rate was sustained at a range of 400–500 beats per minute. In order to assess cardiac contraction and diastolic function, two-dimensional short-axis M-mode echocardiography was conducted. The calculation of aortic PWV involved the acquisition of ascending and abdominal aorta, accompanied by simultaneous electrocardiogram (ECG) recordings. PWV was computed via dividing the distance between two distinct locations by the time delay of two onsets of the pulse wave.

### Statistical analysis

Data are expressed as mean ± SEM. Shapiro–Wilk test was used to evaluate data distribution normality. Student's t-test was used to analyze the two-group comparisons. For comparisons of differences between three or more groups, one-way ANOVA tests followed by Tukey’s posthoc test was used. *P* values of < 0.05 indicate statistical significance.

### Supplementary Information

**Additional file 1. **Supplemental Table and Figures for Targeted Delivery of MerTK Protein via Cell Membrane Engineered Nanoparticle Enhances Efferocytosis and Attenuates Atherosclerosis in Diabetic ApoE-/- Mice.
**Additional file 1: Table S1. **Primers used in the study. **Figure S1. **Diabetes exacerbates atherosclerotic lesions in ApoE-/- mice. **Figure S2. **Effects of diabetes on serum levels of biochemical parameters and arterial stiffness. ** Figure S3. **High glucose changes inflammatory gene expression. **Figure S4. **High glucose damages the efferocytosis capacity in RAW264.7 cells. ** Figure S5. **Increased MerTK expression through viral infection. Figure S6. Characterization of the SMN-Tf.

## Data Availability

The data supporting the finding of this study are availability from corresponding author.
