# Supplementary material for: Targeted delivery of MerTK protein via cell membrane engineered nanoparticle enhances efferocytosis and attenuates atherosclerosis in diabetic ApoE−/− Mice
Source: J Nanobiotechnology. 2024 Apr 13;22:178. doi: 10.1186/s12951-024-02463-y (PMC11015613; doi:10.1186/s12951-024-02463-y)
Supplement: Supplementary file 1 — Additional file 1. Supplemental Table and Figures for Targeted Delivery of MerTK Protein via Cell Membrane Engineered Nanoparticle Enhances Efferocytosis and Attenuates Atherosclerosis in Diabetic ApoE-/- Mice. Additional file 1: Table S1. Primers used in the study. Figure S1. Diabetes exacerbates atherosclerotic lesions in ApoE-/- mice. Figure S2. Effects of diabetes on serum levels of biochemical parameters and arterial stiffness. Figure S3. High glucose changes inflammatory gene expression. Figure S4. High glucose damages the efferocytosis capacity in RAW264.7 cells. Figure S5. Increased MerTK expression through viral infection. Figure S6. Characterization of the SMN-Tf. [file 12951_2024_2463_MOESM1_ESM.pdf]

Additional Information

Targeted Delivery of MerTK Protein via Cell Membrane Engineered Nanoparticle Enhances Efferocytosis and Attenuates Atherosclerosis in Diabetic ApoE$^{-/-}$ Mice

Shuo Qiu[1]†, Jiahan Liu[1]†, Jianmei Chen[2]†, Yangni Li[1], Te Bu[1], Zhelong Li[1], Liang Zhang[1], Wenqi Sun[1], Tian Zhou[1], Wei Hu[1], Guodong Yang[3], Lijun Yuan[1]*, Yunyou Duan[1]*, and Changyang Xing[1]*

1. Department of Ultrasound Medicine, Tangdu Hospital, Air Force Medical University
2. Department of Health medicine, The Fourth Medical Center of Chinese PLA General Hospital
3. The State Key Laboratory of Cancer Biology, Department of Biochemistry and Molecular Biology, Air Force Medical University

† These authors contributed equally to the study.

***Corresponding Authors:**

Changyang Xing

Department of Ultrasound Medicine, Tangdu Hospital, Air Force Medical University,

No.569, Xinsi Road, Xi'an, 710038, China,

E-mail: xingcy@fmmu.edu.cn

Yunyou Duan

Department of Ultrasound Medicine, Tangdu Hospital, Air Force Medical University,

No.569, Xinsi Road, Xi'an, 710038, China,

E-mail: duanyy@fmmu.edu.cn

Lijun Yuan

Department of Ultrasound Medicine, Tangdu Hospital, Air Force Medical University,

No.569, Xinsi Road, Xi'an, 710038, China,

E-mail: yuanlj@fmmu.edu.cn

Table S1. Primers used in the study.

Gene	Forward	Reverse
Il1β	5'GCAACTGTTCCTGAACTCAACT3'	5'ATCTTTTGGGGTCCGTCAACT3'
Nos2	5'GTTCTCAGCCCAACAATACAAGA3'	5'GTGGACGGGTCGATGTCAC3'
Il6	5'TTCCATCCAGTTGCCTTCTT3'	5'CAGAATTGCCATTGCACAAC3'
Tnfα	5'CTGAACTTCGGGGTGATCGG3'	5'GGCTTGTCACTCGAATTTTGAGA3'
Il10	5'GCTCTTACTGACTGGCATGAG3'	5'CGCAGCTCTAGGAGCATGTG3'
Ym1	5'CAGGTCTGGCAATTCTTCTGAA3'	5'GTCTTGCTCATGTGTGTAAGTGA3'
Fizz1	5'CCAATCCAGCTAACTATCCCTCC3'	5'ACCCAGTAGCAGTCATCCCA3'
Ptgs2	5'TGAGCAACTATTCCAAACCAGC3'	5'GCACGTAGTCTTCGATCACTATC3'
Ptges	5'GGATGCGCTGAAACGTGGA3'	5'CAGGAATGAGTACACGAAGCC3'
Mertk	5'CAGGGCCTTTACCAGGGAGA3'	5'TGTGTGCTGGATGTGATCTTC3'
β-actin	5'GGCTGTATTCCCCTCCATCG3'	5'CCAGTTGGTAACAATGCCATGT3'

Additional Figures and Figure legends

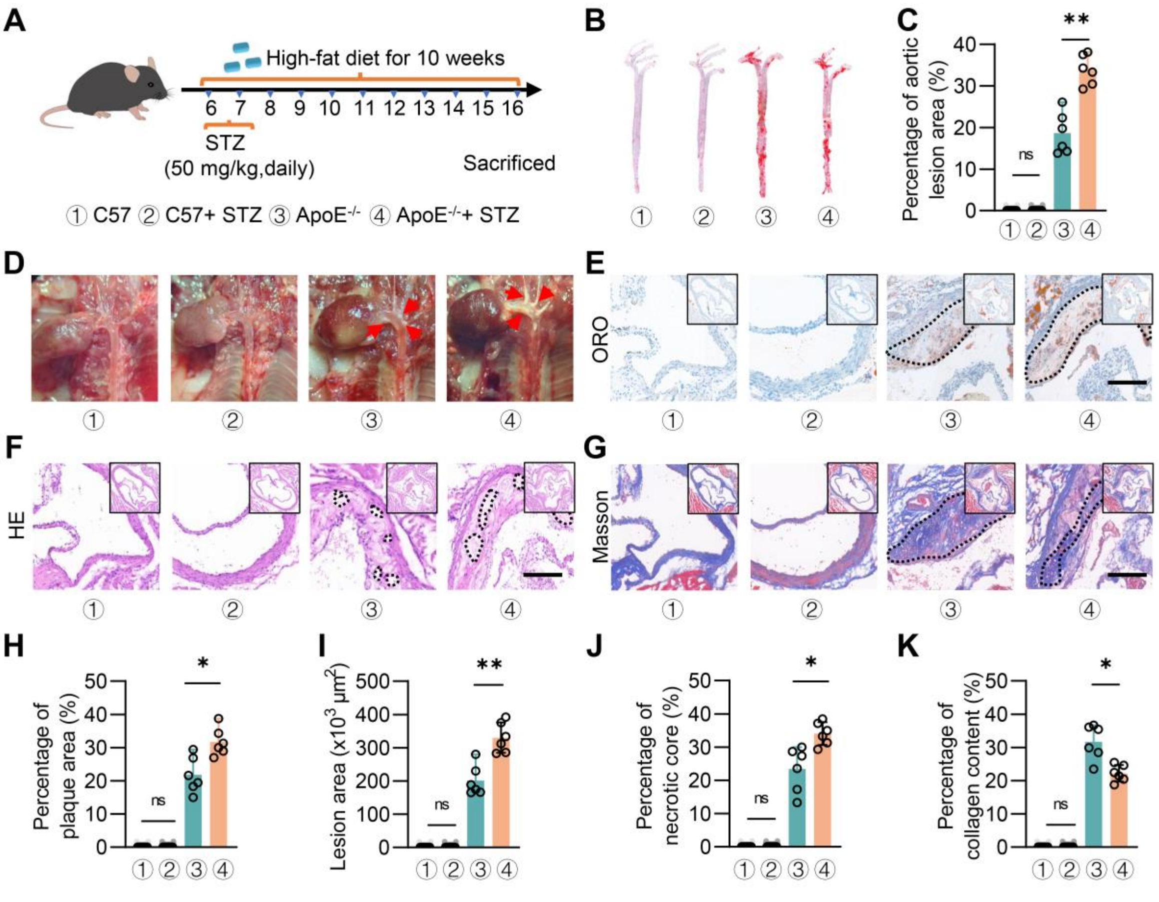

Figure S1. Diabetes exacerbates atherosclerotic lesions in ApoE$^{-/-}$ mice.

A. Schematic diagram showing the experimental procedure. B. Representative images of Oil Red O staining of the atherogenic lesion areas. C. Percentage of the aortic lesion. D. Representative aortic arch view of the atherosclerotic lesions in mice treated as indicated. E-G. Representative images of the atherogenic lesion areas stained with H&E, Masson's trichrome, and Oil Red O. Scale bar = 200 μm. H–K. Quantitative analysis of the aortic lesion percentage, lesion area, necrotic core area, plaque collagen area relative to plaque area. Data are presented as mean ± SEM (n= 6 per group). Statistical significance was determined by one-way ANOVA with Tukey's post hoc test. *P < 0.05, **P < 0.01.

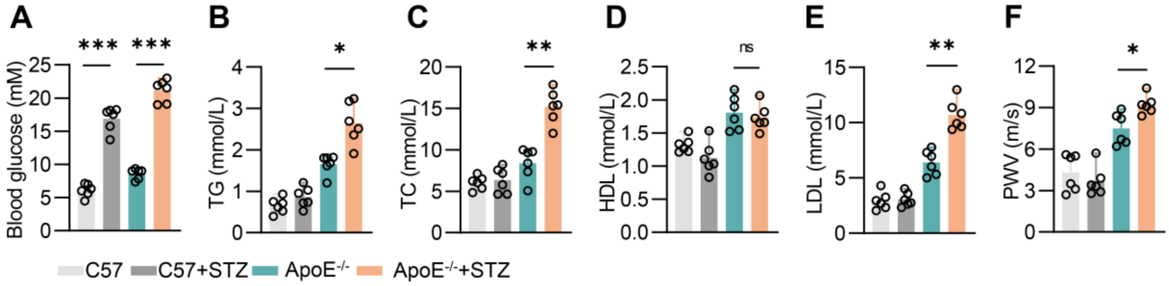

Figure S2. Effects of diabetes on serum levels of biochemical parameters and arterial stiffness.

A-E. Serum biochemistry measured in mice. Examination of the blood glucose, total triglyceride, total cholesterol, HDL, and LDL levels. F. The PWV of the C57, C57+STZ, $ApoE^{-/-}$ and $ApoE^{-/-}$+STZ mice. Data are presented as mean ± SEM (n= 6 per group). Statistical significance was determined by one-way ANOVA with Tukey's post hoc test. *$P < 0.05$, **$P < 0.01$, ***$P < 0.001$. ns, no significance.

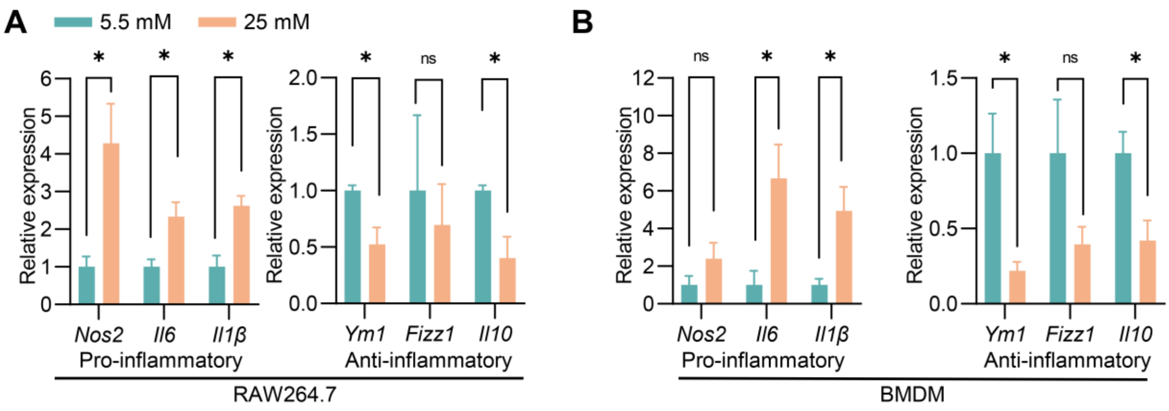

Figure S3. High glucose changes inflammatory gene expression.

RT-qPCR Analysis of pro- and anti-inflammatory cytokine mRNA levels in RAW 264.7 (A) or BMDMs (B). Data are presented as mean ± SEM of three independent experiments. Statistical significance was determined by student's t test, *P < 0.05. ns, no significance.

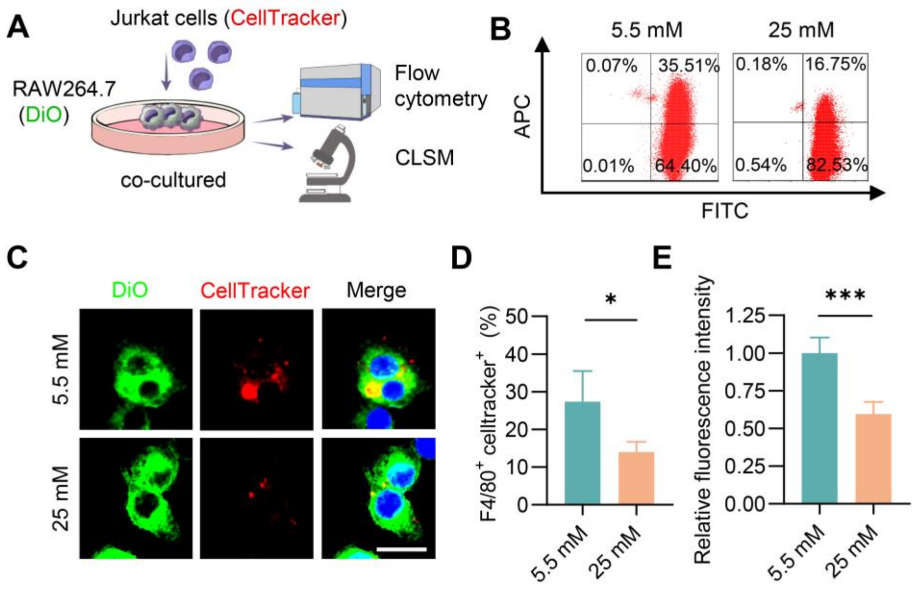

Figure S4. High glucose damages the efferocytosis capacity in RAW264.7 cells.

A. Schematic of efferocytosis assay in vitro. DiO labeled RAW264.7 cells were co-cultured with Celltracker labeled Jurkat cells, followed by immunofluorescence and flow cytometry analysis. B. Flow cytometry analysis of RAW264.7 uptaking apoptotic Jurkat cells. C. Representative confocal laser scanning microscopy (CLSM) showing the localization of apoptotic cells (Jurkat cells) endocytosed by RAW264.7 cells. Scale bar =10 µm. D. Quantitative analysis of flow cytometry data. E. Quantitative analysis of fluorescence intensity. Data are presented as mean ± SEM of three independent experiments. Statistical significance was determined by student's t test, $*P < 0.05$, $***P < 0.001$.

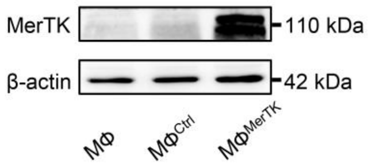

Figure S5. Increased MerTK expression through viral infection.

A. Western blot analysis of MerTK expression in RAW 264.7. Data are presented of three independent experiments.

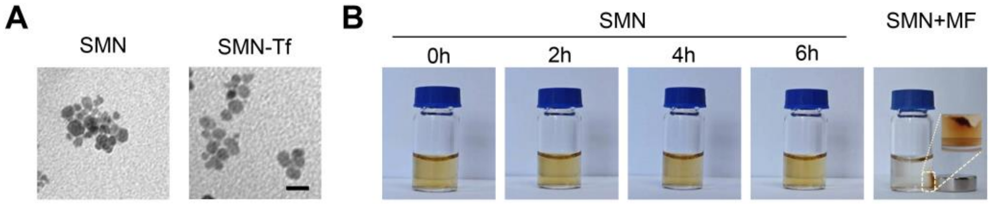

Figure S6. Characterization of the SMN-Tf.

A. Representative TEM images of SMN and SMN-Tf. B. MF induces aggregation of the SMN in vitro. Scale bar = 20 nm. Data are representative of three independent experiments.

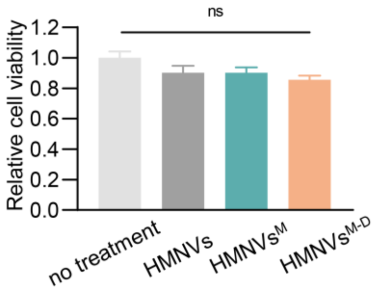

Figure S7. Cell viability in cells with indicated treatments.

Cell viability was measured using cell counting kit-8. Data are presented as mean ± SEM of three independent experiments. Statistical significance was determined by one-way ANOVA with Tukey's post hoc test. ns, not significant.

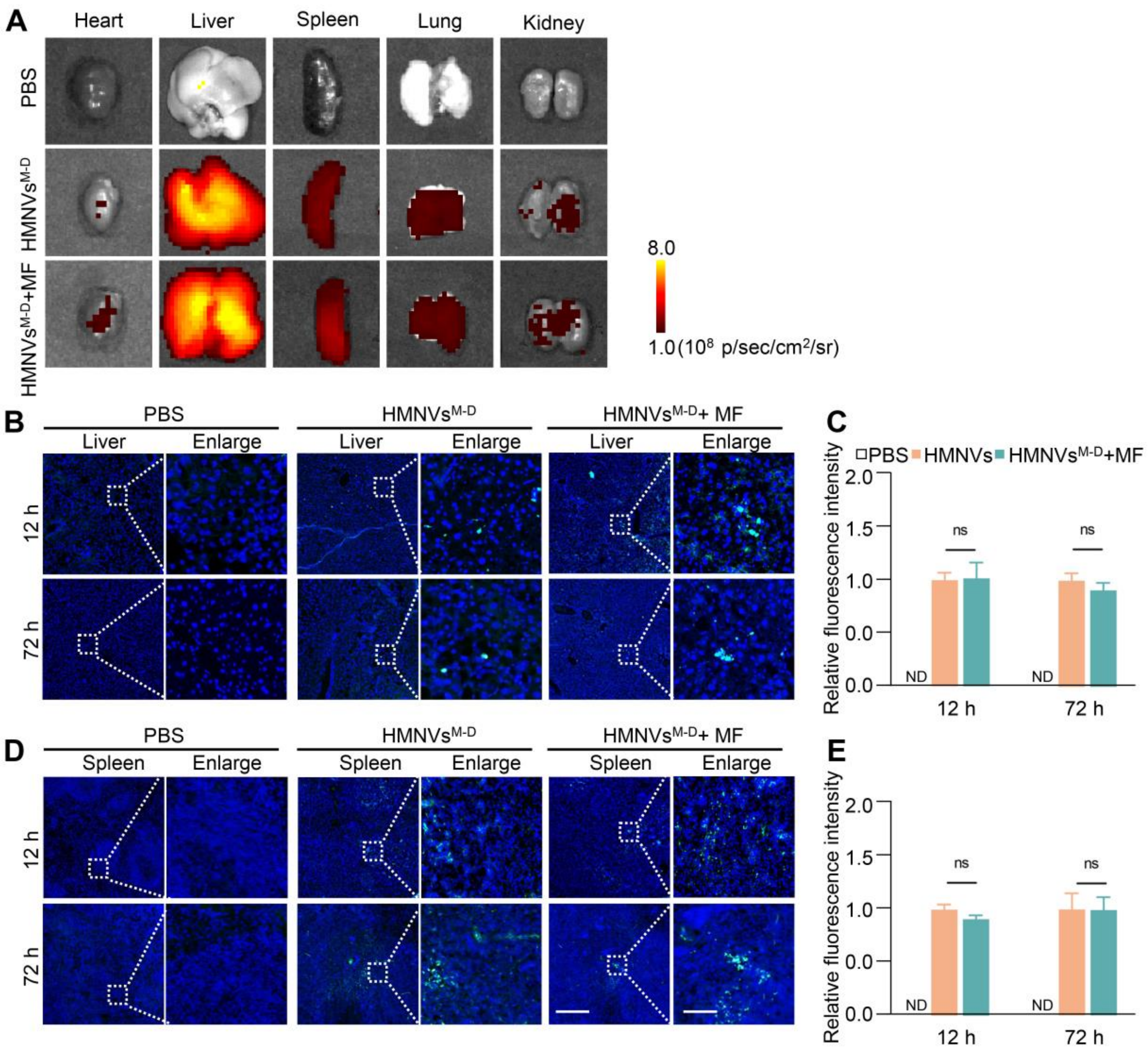

Figure S8. Biodistribution of HMNVs after systemic administration.

A. Quantitative fluorescence analysis of the distribution of the HMNVs in different organs, including heart, liver, spleen, lung, and kidney B. Representative fluorescence images of the HMNVs in the liver (green). The nuclei were counter-stained with Hoechst (blue). C. Quantitative analysis of the fluorescence area. D. Representative fluorescence images of the HMNVs in the spleen (green). The nuclei were counter-stained with Hoechst (blue). Scale bar = 500 or 100 μm. E. Quantitative analysis of the fluorescence area. Data are presented as mean ± SEM (n = 3 per group). Statistical significance was determined by student's t test, ns, no significance.

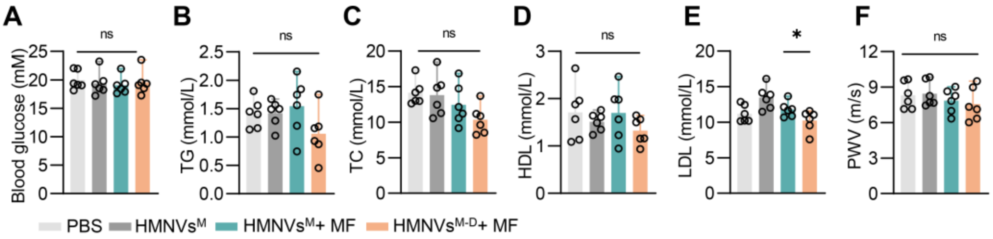

Figure S9. HMNVs attenuate atherosclerotic lesions in diabetic ApoE$^{-/-}$ mice.

A-E. Serum biochemistry measured in mice treated as indicated. Examination of the blood glucose (A), total triglyceride (B), total cholesterol (C), HDL (D), and LDL (E) levels. F. The PWV of mice treated as indicated. Data are presented as mean ± SEM (n= 6 per group). Statistical significance was determined by one-way ANOVA with Tukey's post hoc test. *P < 0.05. ns, no significance.

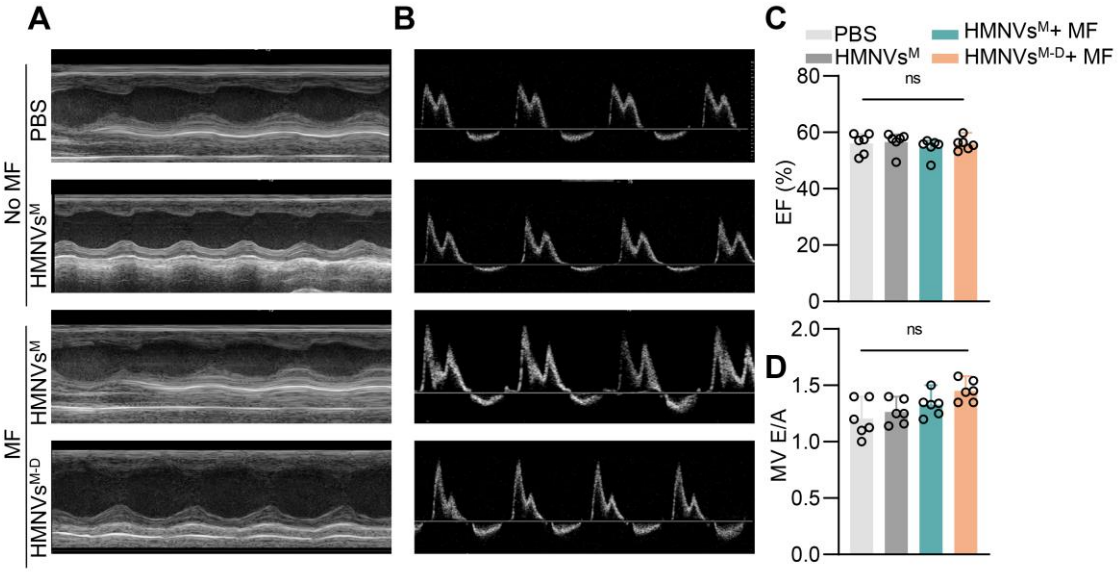

Figure S10. Cardiac function change in mice receiving HMNV treatments. A. Representative images of M mode echocardiography of mice with indicated treatments. B. Representative images of mitral flow Doppler echocardiography. C. Quantification of systolic function parameter ejection-fraction (EF). D. Quantification of diastolic function parameter E/A. Data are presented as mean ± SEM (n= 6 per group). Statistical significance was determined by one-way ANOVA with Tukey's post hoc test. ns, no significance.
